# Clinical outcomes of switching to aflibercept using a *pro re nata* treatment regimen in patients with neovascular age-related macular degeneration who incompletely responded to ranibizumab

**DOI:** 10.1186/s12886-018-0688-3

**Published:** 2018-01-30

**Authors:** Flora Elwes, Shyamanga Borooah, Peter Aspinall, Peng Yong Sim, Cheng Yi Loo, Ana-Maria Armbrecht, Baljean Dhillon, Peter Cackett

**Affiliations:** 1 Royal Infirmary of Edinburgh, Edinburgh, UK; 2 The Princess Alexandra Eye Pavilion, Edinburgh, UK; 3 Health and Environment Group, Heriot Watt University, Edinburgh, UK; 4 College of Medicine & Veterinary Medicine, University of Edinburgh, Edinburgh, UK

**Keywords:** Age-related macular degeneration, Ranibizumab, Aflibercept, Anti-VEGF

## Abstract

**Background:**

To assess the effect of switching patients previously incompletely treated with ranibizumab (RBZ) to aflibercept (AFL) using a *pro re nata* (PRN) treatment strategy in neovascular age-related macular degeneration (nvAMD).

**Methods:**

A retrospective case series was conducted on patients who had persistent or recurrent intra- and/or sub-retinal fluid treated initially with RBZ and subsequently switched to AFL. The main outcome measures were best corrected visual acuity (BCVA) and central retinal thickness (CRT) measured at different stages of the study. Friedman analysis of variance and Wilcoxon test were used to examine differences in BCVA and CRT.

**Results:**

Two hundred and seven eyes from 182 patients were included. BCVA and CRT improved significantly initially following 3 RBZ injections with a mean gain of 3.7 letters (*p* < 0.001) and a mean loss of 69 μm (*p* < 0.001) respectively. Following PRN RBZ therapy and immediately prior to switching to AFL (mean 129 weeks), there was a mean loss of 6.7 letters (*p* < 0.001) BCVA and a mean gain of 24 μm (*p* < 0.001) CRT.

AFL loading resulted in a mean improvement of 0.7 letters (*p* = 0.28) BCVA and 55 μm (*p* < 0.001) CRT. At final follow-up following AFL PRN therapy (mean 85 weeks), there was a mean loss of 8.9 letters (*p* < 0.001) BCVA and a mean gain of 12 μm (*p* < 0.05) CRT.

**Conclusion:**

AFL loading resulted in a significant anatomical improvement but no significant change in visual acuity. However, the benefits of switching were gradually lost over time with AFL PRN dosing despite an increased injection rate when compared with RBZ PRN treatment.

**Trial registration:**

Not applicable

**Electronic supplementary material:**

The online version of this article (10.1186/s12886-018-0688-3) contains supplementary material, which is available to authorized users.

## Background

Neovascular age-related macular degeneration (nvAMD) is one of the leading causes of visual impairment in the population aged over 50 years of age [1]. The prevalence of sight threatening nvAMD is predicted to increase with time [2].

The mainstay of treatment for nvAMD is with intravitreal administration of drugs targeting vascular endothelial growth factor (VEGF). Ranibizumab (RBZ) [Lucentis, Genentech, San Francisco, California, USA] was first licensed by the Federal Drug Administration (FDA) for the treatment of nvAMD in 2006 and became the most widely used anti-VEGF agent. RBZ is a monoclonal antibody fragment that specifically targets VEGF-A [3]. Large multicentre clinical trials have demonstrated that RBZ intravitreal therapy (IVT) stabilises long-term best-corrected visual acuity (BCVA) in the majority of patients with nvAMD and improves BCVA in a minority of patients [4, 5]. However, maintaining a frequent treatment schedule for patients with nvAMD is difficult for patients and places a heavy burden on health services [6]. As a result a number of variable treatment, follow-up and dosing schedules have been developed including *pro re nata* (PRN) strategies [7–9]. Despite RBZ treatment some patients with nvAMD continue to demonstrate persistent macular fluid [10]. Taken together this points to the need to test alternative treatments for nvAMD in patients who are incompletely treated with RBZ IVT.

Aflibercept (AFL) [Eylea, Regeneron, Tarrytown, New Jersey] was FDA-approved as an alternative anti-VEGF treatment for nvAMD in late 2011. AFL is a recombinant fusion protein consisting of VEGF-binding portions from the extracellular domains of human VEGF receptors. These protein domains are fused to the Fc portion of a human immunoglobulin to improve their half-life [11]. AFL mimics VEGF target receptors and acts to trap both VEGF-A, VEGF-B and placental growth factor reducing downstream effects of these chemokines. Multicentre clinical trials have also confirmed the clinical efficacy of AFL in the treatment of nvAMD [12]. Additionally, AFL has also been shown to reduce persistent macular fluid in cases of nvAMD which appear refractory to treatment with RBZ [13, 14]. AFL has been found to have a higher binding affinity for VEGF than RBZ which predicts a lower required concentration and potentially longer clinical effect [15]. AFL administered at an interval of two months after 3 initial monthly loading doses, was found to be non-inferior to RBZ in treatment-naive eyes [12]. The less frequent treatment regime with AFL and reduced cost of AFL compared with RBZ treatment for nvAMD also has potential implications for improved cost-utility when compared with RBZ in a PRN dosing schedule [16].

Increasingly patients who have initially been treated with RBZ are being switched to AFL. However, there is a relative dearth of real world clinical data on the effect of switching patients using a PRN treatment strategy. In this study we describe the effects of switching to AFL PRN therapy in AMD patients who had recurrent nvAMD despite a RBZ PRN schedule at a regional AMD treatment centre in the United Kingdom.

## Methods

### Design & Patients

A retrospective case series was conducted on patients attending the Princess Alexandra Eye Pavilion, Edinburgh, a tertiary referral centre for nvAMD. A treatment register was maintained of all patients who attended the centre between September 2013 and May 2014 and required ongoing anti-VEGF treatment. From this register we identified consecutive patients who were switched from RBZ to AFL. This therapeutic switch was based on a change in protocol for treatment instituted by the department. Switch patients were followed up prospectively for a minimum of 12 months after switch. Paper-based health records for these patients were retrospectively reviewed between 1 April 2016 and 26 June 2016.

Verbal informed consent for prospective data collection was obtained from all participants at the first injection visit by the treating clinician. The study was given ethical approval, granted a waiver of documentation of informed consent for retrospective analysis and approved for verbal consent with regard to prospective follow-up by the NHS Lothian research ethics committee with approval number 09/S1101/05. All procedures adhered to the tenets of the Declaration of Helsinki.

All inclusion criteria refer to the study eye only. In order to be included in the study, patients had to have a diagnosis of nvAMD as demonstrated by changes on retinal examination, optical coherence tomography (OCT) or fundus fluorescein angiography initially. In addition, to be switched they had to have persistent or recurrent intra- and/or sub-retinal fluid on OCT consistent with active disease at least 6 months after RBZ loading. Patients must also have had a minimum of 4 RBZ injections which comprised the loading phase (3 consecutive monthly injections) followed by at least 1 further injection.

Exclusion criteria for the study eye included best-corrected visual acuity ≥1.3 logMAR as this exceeded local guidelines for treatment with anti-VEGF.

### Study protocol

**Fig. 1:**
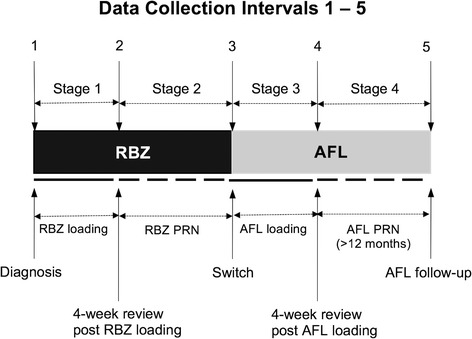
Diagram summarising the timeline of data collection in this study. All patients had an initial loading phase of 3 monthly injections of RBZ (stage 1) followed by a PRN treatment schedule with RBZ (stage 2) which was on average 129 (±86) weeks. After switch, patients had 3 loading monthly injections of AFL (stage 3) followed by PRN AFL (stage 4). The mean time for AFL PRN treatment was 85 (±18) weeks. Data was collected at baseline (1) and 4-week review post RBZ loading (2). Further data was collected at switch to AFL (3), 4-week review post AFL loading (4) and > 12 months after switch (5)

For all patients, data was collected at 5 intervals representing 4 stages of the study (Fig. 1). In stage 1, patients were reviewed prior to initiating RBZ treatment and 4 weeks after the third loading dose of RBZ. In stage 2, patients were then treated with RBZ following a PRN protocol.

At switch, 3 loading doses of intravitreal AFL (2 mg) were administered at approximately 4-week intervals. Stage 3 was from immediately prior to anti-VEGF switch and 4 weeks after the third loading dose of AFL. In stage 4 patient were treated with AFL again on a PRN schedule and followed up after a minimum of 12 months following AFL switch. For both PRN phases, a decision to retreat was made if there was evidence of intra- and/or sub-retinal fluid on SD-OCT.

At all visits, patients underwent a full ophthalmic examination of both eyes, which included best corrected visual acuity (BCVA) measured using Early Treatment Diabetic Retinopathy Study (ETDRS) letters (recorded at 4 m) whilst central retinal thickness (CRT) measured using OCT. The same Zeiss Cirrus SD-OCT machine (Carl Zeiss Meditec Inc., Dublin, CA) was used for all CRT measurements. Additionally, intraocular pressures and adverse event monitoring was performed at each visit. Data was also collected on the duration of disease, the number of injections performed and phakic status. If follow-up was deemed appropriate, the schedules were decided by the ophthalmologist depending on activity and varied between 1 and 4 months.

### Injection procedure

All RBZ and AFL injections were performed according to a standardised protocol in a treatment room setting by the same healthcare team. Patients were prepared by administration of topical anaesthesia (Minims tetracaine hydrochloride 0.5%), followed by application of a topical antiseptic (povidine iodine 5% or chlorhexidine 0.1% in the case of documented prior iodine allergy). The injection was performed using a 30-gauge needle through the *pars plana*. All patients were given a one-off dose of chloramphenicol ointment following injection.

### Statistical analysis

The analysis was performed in three parts. Firstly, differences in BCVA and CRT were examined using a Friedman analysis of variance followed by Wilcoxon tests for paired comparisons. Secondly, correlations between change in BCVA and CRT were analysed by principle component analysis with varimax rotation. Finally, a correlated component regression analysis examining the predictors of change in visual outcome was carried out at each of the 4 stages of the study [see Additional file 1 for further details of statistical analyses]. All statistical analyses were carried out using SPSS statistic version 17.0 (IBM Corp., Armonk, N.Y., USA).

## Results

### Patient characteristics

A total of 213 eyes from 188 patients were identified from the treatment register; 6 of the 188 patients were excluded due to missing case notes, leaving 207 eyes from 182 patients for analysis. A summary of the baseline details of the participants at switch and duration of AMD by switch and number of RBZ injections is shown in Table 1. The mean age at switch was 80 years (±7.7) and 71% of patients were female. The mean duration of nvAMD in patients was 129 (±86) weeks at switch. Baseline mean BCVA (±SD) was 29 (±13.2) ETDRS letters whilst the baseline mean CRT (±SD) was 342 μm (±100).

**Table 1:** Summary of baseline patient details

Characteristics	
No. of participants	182
No. of eyes	207
Mean age (years) ± SD	80 ± 7.7
Female (%)	71
Phakic eyes (%)	78
Mean BCVA (letters) ± SD	29 ±13.2
Mean central retinal thickness (μm) ± SD	342 ± 100
Mean duration of nvAMD at switch (weeks) ± SD	129 ± 86
Mean number of ranibizumab injections at switch ± SD	12 ± 7

Patients received monthly RBZ injections for RBZ loading in stage 1. The mean treatment period during stage 2 (RBZ PRN) was 129 (±86) weeks with patients receiving an average of 12 (±7) RBZ injections over this time.

At switch patients received 3 loading doses of AFL with a month interval. In stage 4, patients received AFL PRN therapy. 9 patients were lost to follow-up, 7 patients were swapped back to RBZ due to poor response to AFL, and a further 7 patients did not require further anti-VEGF treatment after AFL loading but continued under regular follow-up. These latter two groups of patients are included in the overall analysis as well as a separate analysis within this paper. The average follow-up for stage 4 of the study was 85 (±18) weeks with patients receiving an average of 10 (±3) AFL injections over this time, achieving an average injection frequency of 8.6 weeks.

### Visual acuity changes

BCVA improved significantly following 3 RBZ loading injections, with a mean gain of 3.7 ETDRS letters (*p* < 0.001) when compared to baseline. At switch, BCVA had deteriorated with a mean loss of 6.7 letters (*p* < 0.001). This was a net loss of 3 ETDRS letters from baseline after a mean treatment period of 129 weeks with a mean of 12 injections resulting in a mean injection frequency of an injection every 10.75 weeks.

**Fig. 2:**
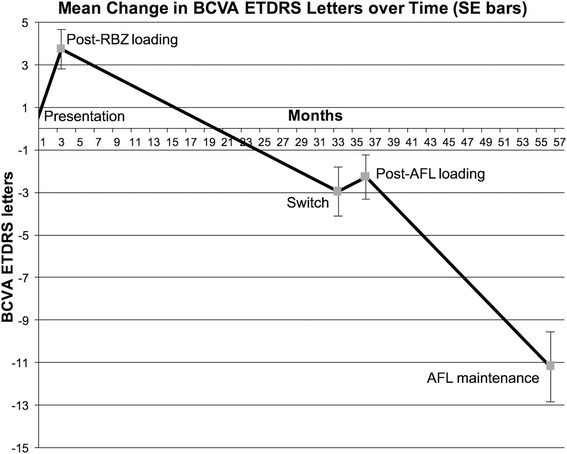
Change in ETDRS letters with standard error bars

After AFL loading, the BCVA had increased by a mean of 0.7 EDTRS letters from switch, although this change (*p* = 0.28) was not statistically significant from BCVA at switch. After the AFL PRN treatment phase mean BCVA had declined with a mean loss of 8.9 letters (*p* < 0.001) over a mean treatment phase of 85 (±18) weeks (Fig. 2). This resulted in an overall mean loss of 11.2 letters from baseline with a total mean treatment time of 229 weeks. The rate of decline of BCVA during RBZ PRN treatment saw an average loss of 3.2 letters per eye per year. In the AFL PRN treatment phase BCVA was reduced on average by 5.5 letters per eye per year. Letters per eye per year was defined as the total sum of letters gained or lost divided by the total number of eyes and the total mean treatment time of the stage examined.

### Anatomic changes

**Fig. 3:**
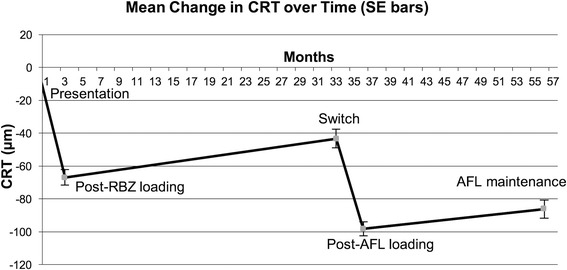
Change in central retinal thickness with standard error bars

Changes in CRT followed a similar trend to BCVA. CRT decreased by an average of 69 μm (*p* < 0.001) following the 3 RBZ loading injections. At switch CRT had increased by a mean of 24 μm (*p* < 0.001). Following AFL loading, the CRT showed a significant mean reduction of 55 μm (*p* < 0.001). After AFL PRN treatment, the CRT had increased by an average of 12 μm (*p* < 0.05) (Fig. 3).

Looking at average changes over time per eye per year for each stage, with RBZ PRN therapy there was an increase in mean CRT of 12 μm per eye per year, which was reduced to 7 μm per eye per year on AFL PRN.

After AFL loading, 173 eyes (83.6%) showed static or reduced CRT. However, after a minimum of 12 months follow-up of patients on AFL PRN treatment only 80 eyes had static or reduced CRT (43%) (Table 2).

**Table 2:** CRT changes following AFL PRN treatment

CRT reduced	N (%)	CRT increased	N (%)
≥50μm	23 (12%)	≥50μm	38 (21%)
≥100μm	8 (4%)	≥100μm	16 (9%)

A principal component analysis with varimax rotation was carried out to examine the relationship between BCVA and CRT during the study. The results showed that there was a positive correlation between BCVA and CRT change in stage 1, meaning that better BCVA was associated with reduced CRT during RBZ loading. However, this relationship was not maintained in stages 2, 3 or 4.

To identify predictors of final visual outcome a regression analysis was used to examine the predictors of change in BCVA and CRT at each stage of the study. Better BCVA at the start of stages 1, 2 and 3 was the only consistent predictor of better BCVA at the end of each of these stages. In stage 4, higher CRT at switch and higher CRT post AFL loading predicted better BCVA. A longer time from first presentation to switch predicted better BCVA after AFL loading whilst a longer time on RBZ PRN therapy predicted worse BCVA after AFL loading. The number of injections did not predict BCVA at any stage. Predictors of CRT included the CRT at the beginning of each stage. Higher final CRT was also predicted by higher CRT and better BCVA post AFL loading and by worse BCVA and lower CRT after RBZ loading [see Additional file 1].

### RBZ PRN vs. AFL PRN

A comparable trend of disease progression was observed following PRN therapy for both RBZ and AFL. There was a mean loss of 6.7 letters (*p* < 0001) BCVA and a mean gain of 24 μm (*p* < 0.001) after RBZ PRN therapy. Similarly, there was a mean loss of 8.9 letters (*p* < 0.001) BCVA and a mean gain of 12 μm (*p* < 0.05) CRT following PRN treatment with AFL PRN therapy.

In order to investigate whether response to RBZ PRN treatment correlated with AFL PRN treatment, we categorised the change in CRT and BCVA during the RBZ PRN treatment phase into different groups [see Additional file 2]. Spearman’s rank-order correlation coefficient revealed no significant correlation between these different groups with respect to change in CRT or BCVA during AFL PRN, suggesting no significant link between RBZ response and AFL response in our patient cohort.

### Data on patients having both eyes treated

A comparison of the data on 25 patients who had both eyes treated was made with the data from the main study. The purpose of this was to investigate if the BCVA and CRT changes across this group were similar to those in the main study. We also assessed for any correlation between the data from the two eyes. In summary, changes in BCVA and CRT for the ‘both eye’ group were found to be similar to those in the main study. We found no significant correlation between the right eye and left eye data for this group [see Additional file 3].

### Switch-back data

Out of 207 study eyes switched to AFL, 7 were switched back to RBZ after AFL loading due to clinical worsening of intra- or sub-retinal fluid. At switch, they had received an average of 15 RBZ injections over a treatment period of 2.2 years. After AFL loading they had a mean loss of 3.8 ETDRS letters and mean increase of 25 μm CRT. At the final data collection, these patients had been followed-up for an average of 23 months after switch. By this time, they had received an average of 4.7 AFL injections and 25 RBZ injections. Compared to post-AFL loading results they had lost an average of 4.6 ETDRS letters with no change in mean CRT.

### Data on patients not receiving any further IVT injections

Following 3 AFL loading injections, 7 patients continued under regular follow-up but received no further anti-VEGF injections. Prior to switch they had received an average of 6.6 RBZ injections. At the final data collection, these patients had an average CRT of 213 μm and average ETDRS score of 56 letters. Compared to results collected after AFL loading this represented a CRT reduction of 34 μm and a loss of 5 ETDRS letters.

### Adverse events

Adverse events were monitored during the period of AFL treatment and are outlined in Table 3. There were no cases of endophthalmitis.

**Table 3:** Adverse events reported during the study

Characteristics	Frequency
Significant loss in VA (progressive retinal scarring)	2
Epithelial defect secondary to intravitreal injection	1
Subretinal haemorrhage	1
Subconjunctival haemorrhage	1
Retinal tear requiring retinopexy	1
Atrial fibrillation	1
Stroke	1
VZV infection/Shingles	2
Metastatic cancer diagnosed during treatment	1

## Discussion

This study compared visual and anatomical outcomes in nvAMD patients who already appeared to have persistent or recurrent nvAMD on RBZ IVT and subsequently transitioned to AFL using a PRN dosing schedule after an initial loading period.

In clinical trials, AFL has been shown to have a similar efficacy and side effect profile to RBZ but with a longer dosing interval [17]. It has been suggested that AFL may be more effective in cases of refractory disease following preclinical studies which have shown that VEGF Trap-Eye binds to VEGF-A with a higher affinity than other anti-VEGF molecules [18]. Additionally, it also acts on other molecules that may result in intra- or sub-retinal fluid such as placental growth factor (PIGF) which are not inhibited by other anti-VEGF drugs [18]. We found that a small number of patients (*n* = 7) did not require any further treatment after initial AFL loading. However, an equal number were also switched back to RBZ due to worsening of intra- and/or sub-retinal fluid.

The outcomes in this study show that initial visual gains made by RBZ therapy on treatment-naive eyes are gradually lost over time using a PRN schedule of treatment. In our study, BCVA and CRT showed significant correlation in stage 1 but not in stages 2, 3 or 4. This suggests that at early stages BCVA may mainly be affected by intra- or sub-retinal fluid which is reflected by CRT. However, at later stages the CRT reflects remaining neuroretinal thickness rather than intra- or sub-retinal fluid. Consequently, higher CRT should lead to better vision. This is supported by findings in stage 4 of our study which found that higher CRT post AFL loading predicted better final BCVA. We did not measure neuroretinal thickness separately in this study. Similar results of a disparity in CRT and BCVA have been found in long term studies with one anti-VEGF agent alone using PRN dosing [10, 19–21] and following transitioning between anti-VEGF agents [22].

A recent systematic review on patients with treatment-resistant nvAMD on bevacizumab or RBZ subsequently switched to AFL concluded that switching led to significant improvement in CRT but only static BCVA [23]. In contrast, we found that there was gradual deterioration in CRT and BCVA with AFL PRN therapy following an initial improvement with AFL loading. This difference in outcomes might be explained by the longer mean follow-up period (24 months) after AFL switch in our study compared to that of the studies (6–12 months) included by Spooner et al. [23] in their review. A likely interpretation is that a follow-up period of 12 months might not be long enough to capture the full long-term effects of switching to AFL.

There has already been some evidence that outcomes in routine clinical practice for new medications do not match the outcomes found in clinical trials where there are stringent inclusion and exclusion criteria and strict follow-up schedules [24, 25]. A potential cause of the deterioration of vision in stage 2 and the subsequent non-significant improvement of vision and significant improvement in CRT after AFL loading could be under treatment. In this study, patients were treated using a PRN protocol. However, this study compares well with recently published PRN treatment clinical trials when comparing numbers of injections per eye per year. The PrONTO study applied 9.9 injections per eye over two years using OCT guided increase in fluid as a guide to retreatment [7]. This approximates to 0.41 RBZ injections per eye per month. A comparable 0.37 RBZ injections were performed per eye per month in this study. Additionally, we did not find that the number of injections predicted better outcomes in the analysis of this study which suggests that the patients were not significantly undertreated.

There is increasing evidence that tachyphylaxis with anti-VEGF agents may play a part in reduction of clinical efficacy with time [26, 27]. In our study, some of the effect may also be a result of tachyphylaxis to RBZ and the subsequent benefit of switching anti-VEGF agents. It is unclear whether a change to another anti-VEGF agent such as bevacizumab would also have resulted in similar short term improvements as has been reported elsewhere [28]. Tachyphylaxis to AFL treatment may also explain the progressive worsening of visual acuity and CRT following longer term treatment with AFL in stage 4 [29]. In our study only seven patients were reverted back to RBZ due to clinical failure of AFL. It would be interesting to see the effects on CRT and BCVA of switching back more patients to RBZ in a future study in order to estimate the effect of tachyphylaxis on clinical response.

A limitation of our study may be the retrospective review of notes to obtain data. The retrospective data relies on the quality of record keeping to ensure accuracy of the data and may particularly affect history, examination findings and BCVA. Nonetheless, measurements were taken at each visit and as a result an objective measure of CRT was obtained for each time point.

Long-term data comparing RBZ and AFL injection frequency has suggested a reduced required injection frequency with AFL using a treat and extend protocol [30]. However, we found that the number of injections required for treatment increased with an AFL PRN treatment regime. 0.47 AFL injections per eye per month were performed compared to 0.37 RBZ injections per eye per month. Our study is unable to provide direct comparison of injection rates as no control group was used and AFL treatment was performed on those previously treated with RBZ and not on treatment naïve patients. Despite the increased injection rate with AFL we continued to notice a decline in visual acuity again confirming that a degenerative process may have already started prior to switch which could not be halted by AFL therapy. A future study plans to examine a cost-benefit analysis of transitioning patients to AFL on a PRN basis as we found that there was an increase in the number of injections required with AFL which may negate the possible cost savings reported [22].

## Conclusion

In summary, this study finds that in patients with nvAMD who are treated with PRN RBZ therapy, macular anatomy is significantly improved following AFL loading. However, this real world clinical data shows that, in the longer term, AFL does not halt the slow deterioration in BCVA and CRT which occurs in patients switched from RBZ despite a higher injection rate using PRN AFL dosing.

## Additional files

Additional file 1: Supplementary data. Correlated component regression analysis examining the predictors of change in BCVA or CRT at each of the 4 stages of the study. (PDF 38 kb)

Additional file 2: Supplementary data. Subgroup analysis of patients who incompletely responded to RBZ based on the extent of response to RBZ PRN therapy. (PDF 23 kb)

Additional file 3: Supplementary data. Analysis of patients having both eyes treated. (PDF 17 kb)

## Abbreviations

AFL: Aflibercept
BCVA: Best-corrected visual acuity
CRT: Central retinal thickness
EDTRS: Early Treatment Diabetic Retinopathy Study
FDA: Federal Drug Administration
IVT: Intravitreal therapy
nvAMD: Neovascular age-related macular degeneration
OCT: Optical coherence tomography
PRN: *Pro re nata*
RBZ: Ranibizumab
VEGF: Vascular endothelial growth factor
