# Supplementary material for: Clinical outcomes of switching to aflibercept using a pro re nata treatment regimen in patients with neovascular age-related macular degeneration who incompletely responded to ranibizumab
Source: BMC Ophthalmol. 2018 Jan 30;18:20. doi: 10.1186/s12886-018-0688-3 (PMC5789603; doi:10.1186/s12886-018-0688-3)
Supplement: Supplementary file 1 — Supplementary data. Correlated component regression analysis examining the predictors of change in BCVA or CRT at each of the 4 stages of the study. (PDF 38 kb) [file 12886_2018_688_MOESM1_ESM.pdf]

Additional File 1 (Supplementary data)

Each stage (stages 1 to 4) was modelled for predictors of change in BCVA or CRT in each stage.

1. BCVA

a) Stage 1: Dependent BCVA at end of stage has one predictor

	Training	Cross-Validation	Std Error
R squared	0.18	0.15	0.01
AUC	0.74	0.74	0.01
Accuracy	0.69	0.68	0.01
		Std Coefficient	Out of sample Freq (n=200)
Predictors			
BCVA baseline		2.14	200

b) Stage 2 – Dependent BCVA at end of stage has one predictor

	Training	Cross validation	Std error
Rsquared	0.05	0.02	0.01
AUC	65	61	0.01
Accuracy	76	75	0.01
		Std Coeff	Out of sample Freq (n=200)
Predictors			
BCVA at start of stage 2		0.77	200

c) Stage 3 - Dependent BCVA at end of stage has 3 predictors. These are days to switch and 1st injection to switch, and BCVA at start of stage

	Training	Cross validation	St error
Rsquared	0.07	0.02	0.01
AUC	0.65	0.57	0.01
Accuracy	0.62	0.59	0.01

		Std Coeff	Out of sample Freq (n=200)
Predictors	Pratt Importance In Model %		
Days from RBZ loading to switch	58	- 3.4	200
Days from 1st presentation to switch	29	1.9	192
BCVA at start of stage 3	13	0.76	189

d) Stage 4 - Dependent BCVA at end of stage has two predictors: CRT after AFL loading, CRT at switch

	Training	Cross validation	St error
Rsquared	0.07	0.02	0.01
AUC	0.67	0.60	0.01
Accuracy	0.78	0.76	0.01
		Std Coeff	Out of sample Freq (n=200)
Predictors	Pratt Importance in model %		
CRT post AFL loading	57	0.46	200
CRT switch	43	0.43	199

2. CRT

a) Stage 1: Dependent CRT at end of stage had one predictor: initial CRT

	Training	Cross-Validation	Std Error
R squared	0.15	0.03	0.04
AUC	0.83	0.77	0.02
Accuracy	0.81	0.80	0.01
		Std Coefficient	Out of sample Freq (n=200)
Predictors			
CRT baseline		4.6	198

b) Stage 2: Dependent CRT change had one predictor: initial CRT at start of stage 2

	Training	Cross-validation	Std error
Rsquared	0.14	0.1	0.01
AUC	0.74	0.71	0.01
Accuracy	0.69	0.68	0.02
		Std Coefficient	Out of sample freq (n=200)
Predictors			
CRT post RBZ loading		1.64	200

c) Stage 3: Dependent CRT at end of stage has one predictor: CRT at switch

	Training	Cross validation	Std error
Rsquared	0.16	0.1	0.04
AUC	0.82	0.76	0.02
Accuracy	0.87	0.84	0.01
		Std Coefficient	Out of sample Freq(n=200)
Predictors			
CRT at switch		4.9	196

d) Stage 4: Dependent CRT at end of stage had four predictors: initial CRT and BCVA at beginning of stage and BCVA and CRT post RBZ loading

	Training	Cross validation	Std Error
Rsquared	0.13	0.03	0.02
AUC	0.69	0.56	0.02
Accuracy	0.65	0.63	0.01
		Std Coefficient	Out of sample freq (n=200)
Predictors	Pratt importance in model %		
CRT post AFL loading	59	1.53	199
BCVA post AFL loading	25	1.22	197
CRT post RBZ loading	8	- 0.78	182
BCVA post RBZ loading	9	- 0.89	173

Note in the above that a 7 predictor optimum at AUC=0.58 (se=0.02) reduces to 4 predictor on standard error rule where AUC=0.560 and contribution to model
