# Supplementary material for: Clinical outcomes of switching to aflibercept using a pro re nata treatment regimen in patients with neovascular age-related macular degeneration who incompletely responded to ranibizumab
Source: BMC Ophthalmol. 2018 Jan 30;18:20. doi: 10.1186/s12886-018-0688-3 (PMC5789603; doi:10.1186/s12886-018-0688-3)
Supplement: Supplementary file 2 — Supplementary data. Subgroup analysis of patients who incompletely responded to RBZ based on the extent of response to RBZ PRN therapy. (PDF 23 kb) [file 12886_2018_688_MOESM2_ESM.pdf]

Additional File 2 (Supplementary Data)

Summary statistics for CRT and BCVA change in patients following RBZ PRN

CRT

**Description**	**Change in CRT (μm)**	**No. of eyes**
Decrease (i.e. improvement) in CRT following RBZ PRN	1 – 50	44
	51– 100	16
	>100	5
No change	0	11
Increase (i.e. deterioration) in CRT following RBZ PRN	1 – 50	38
	51– 100	28
	>100	31
Total of those with complete CRT data		173
Missing CRT readings (either pre- or post RBZ PRN)		35
**Total**		**208**

Spearman rank-order correlation coefficient revealed no significant correlation between these different groups with CRT (p=0.268, r= –0.085) or BCVA (p=0.373, r= –0.068) during AFL PRN.

BCVA

**Description**	**Change in letters**	**No. of eyes**
Letters gained (i.e. improvement in BCVA) following RBZ PRN	1 – 5 (1)	31
	6 – 10 (2)	14
	11 – 15 (3)	8
	>15 (4)	2
No change	0 (5)	21
Letters lost (i.e. deterioration in BCVA) following RBZ PRN	1 – 5 (6)	43
	6 – 10 (7)	28
	11 – 15 (8)	23
	>15	38
Total of those with complete BCVA data **(no missing data)**		**208**

Similarly, Spearman rank-order correlation coefficient revealed no significant correlation between these different groups with CRT (p=0.935, r= 0.006) and or BCVA (p=0.072, r=0.126) during AFL PRN.
