# Supplementary material for: Clinical outcomes of switching to aflibercept using a pro re nata treatment regimen in patients with neovascular age-related macular degeneration who incompletely responded to ranibizumab
Source: BMC Ophthalmol. 2018 Jan 30;18:20. doi: 10.1186/s12886-018-0688-3 (PMC5789603; doi:10.1186/s12886-018-0688-3)
Supplement: Supplementary file 3 — Supplementary data. Analysis of patients having both eyes treated. (PDF 17 kb) [file 12886_2018_688_MOESM3_ESM.pdf]

Additional File 3 (Supplementary Data)

A comparison of the data on 25 patients who had both eyes treated was made with data from the main study. The purpose of this was to investigate if the BCVA and CRT changes across this group were similar to those in the main study and in addition whether there were correlations between the data from the two eyes.

Firstly the changes in BCVA and CRT across the two eye data were found to be similar to those in the patients in the main study. For example, on a Friedman test a significant difference in BCVA across the 4 sampling points was found ($\chi 2$=13.1, df=3, p=0.004), and there were also significant differences across CRT ($\chi 2$= 27.9, df=3, p=<0.001). In addition the difference tests on right eyes and left eyes separately showed a similar order of magnitude ($\chi 2$=7.8 for right eyes and $\chi 2$=6.8 for left eyes) with p values borderline at p=0.05 and p=0.07 respectively as a consequence of the reduced sample size.

Secondly two separate Spearman correlations between right eye and left eye data for the initial and the final BCVA changes were found to be not significant (r=0.14, p=0.53 for initial BCVA, and r=0.27, p=0.20 for final BCVA).
